# Nesting-driven multipolar order in CeB_6_ from photoemission tomography

**DOI:** 10.1038/ncomms10876

**Published:** 2016-03-15

**Authors:** A. Koitzsch, N. Heming, M. Knupfer, B. Büchner, P. Y. Portnichenko, A. V Dukhnenko, N. Y. Shitsevalova, V. B. Filipov, L. L. Lev, V. N. Strocov, J. Ollivier, D. S. Inosov

**Affiliations:** 1 Institute for Solid State Research, IFW-Dresden, PO Box 270116, D-01171 Dresden, Germany; 2 Institut für Festkörperphysik, TU Dresden, D-01069 Dresden, Germany; 3 I.M. Frantsevich Institute for Problems of Materials Science of NAS, 3 Krzhyzhanovsky str., Kiev 03680, Ukraine; 4 Paul Scherrer Institut, Swiss Light Source, CH-5232 Villigen PSI, Switzerland; 5 National Research Centre Kurchatov Institute, 123182 Moscow, Russia; 6 Institut Laue-Langevin, 6 rue Jules Horowitz, BP 156, 38042 Grenoble Cedex, France

## Abstract

Some heavy fermion materials show so-called hidden-order phases which are invisible to many characterization techniques and whose microscopic origin remained controversial for decades. Among such hidden-order compounds, CeB_6_ is of model character due to its simple electronic configuration and crystal structure. Apart from more conventional antiferromagnetism, it shows an elusive phase at low temperatures, which is commonly associated with multipolar order. Here we show that this phase roots in a Fermi surface instability. This conclusion is based on a full 3D tomographic sampling of the electronic structure by angle-resolved photoemission and comparison with inelastic neutron scattering data. The hidden order is mediated by itinerant electrons. Our measurements will serve as a paradigm for the investigation of hidden-order phases in *f*-electron systems, but also generally for situations where the itinerant electrons drive orbital or spin order.

## Abstract (web summary)

In compounds containing 4*f* and 5*f* elements, hidden-order phases exist which are undetectable by many methods, the origins of which are debated. Here, the authors use photoemission and neutron scattering methods to show how such a multipolar-ordered phase emerges due to Fermi surface instability in CeB_6_.

## Graphical abstract

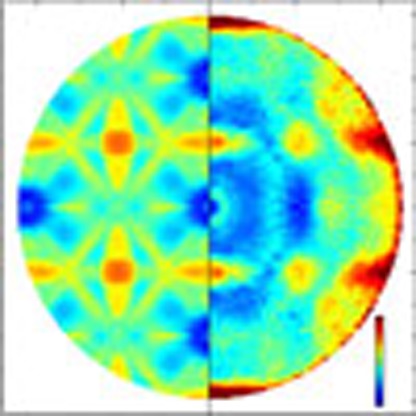

Hidden-order phases have been observed in a variety of compounds containing 4*f* and 5*f* elements, for example, URu_2_Si_2_ (ref. 1), NpO_2_ (ref. 2), skutterudites^3^ and YbRu_2_Ge_2_ (ref. 4). They are characterized by a rich low-temperature phase diagram and it is assumed that the multipolar moments of the *f*-electrons in their specific crystal field environment play a decisive role^5^,^6^.

CeB_6_ is a heavy-fermion material showing a mass enhancement of the order of 100 (ref. 7), which is due to the hybridization of the localized *f*-electrons with the itinerant conduction electrons. Magnetism of heavy fermion materials is determined by the competition of Kondo screening and the Ruderman–Kittel–Kasuya–Yosida (RKKY) interaction, the former quenching the local moments and favouring paramagnetic behaviour, the latter promoting magnetic order mediated by the conduction electrons^8^,^9^. In CeB_6_ the usual paramagnetic response is found before antiferromagnetic order with a double-**Q** commensurate structure characterized by the propagation vectors **Q**_AFM1_=(*π*/2, *π*/2, 0) and **Q**_AFM2_=(*π*/2, *π*/2, *π*) sets in below *T*_N_=2.3 K. However, the phase diagram is more complex: the antiferromagnetism is preceded by a famous hidden order state at *T*_Q_=3.2 K, the so called antiferroquadrupolar phase (AFQ), which has been explained by the ordering of quadrupole moments with **Q**_AFQ_=(*π*, *π*, *π*)^10^,^11^. The latter has long been elusive to neutron diffraction experiments and was first directly visualized by X-ray scattering^12^.

Attempts were made to describe these observations by theories emphasizing the local character of the magnetic moments^13^,^14^. However, recently a magnetic resonance mode has been discovered at **Q**_AFQ_ below *T*_N_ in close resemblance to the ones in unconventional superconductors^15^, underlining the importance of itinerant spin dynamics for this compound.

In an itinerant picture, the strength of the magnetic interactions is mediated by the conduction electrons and depends on the low-energy electronic structure. It can be expressed within linear response theory by the Lindhard function^16^. The latter quantifies the propensity of a given electronic structure towards nesting instabilities of the Fermi surface and the subsequent formation of a new, in our case magnetically ordered, state^17^. An interesting question in this context is whether or not the AFQ state also is directly linked to the electronic structure in a similar way. It has been theoretically suggested that the interactions between multipolar moments in CeB_6_ is driven by a RKKY-type interaction^18^. However, although CeB_6_ has been studied for more than 50 years^19^, the three-dimensional (3D) electronic structure of CeB_6_ was not known so far neither from experiment nor from theory with sufficient accuracy to test this hypothesis. This deficiency calls for a detailed investigation of the band structure and the Fermi surface of CeB_6_.

Here we implement a rigorous and innovative approach: We measured samples cleaved along all high-symmetry crystallographic planes (100), (110), (111). This probes different planes of **k**-space, resembling a tomographic type of measurement that yields complete 3D information about the electronic structure in contrast to conventional angle-resolved photoemission spectroscopy (ARPES), in which one direction orthogonal to the surface is always inferior to two others. We conducted photon-energy dependent measurements in the soft X-ray regime spanning a wide *k*_*z*_ interval^20^. In comparison with conventional low-energy ARPES, this increases the photoelectron mean free path *λ*, which in turn increases the signal of the bulk states^21^ and, crucial for 3D materials like CeB_6_, enhances the intrinsic *k*_*z*_ resolution^22^. From the consistency of the results obtained in this way, we can infer the absence of surface-related effects. Moreover, such a full data set offers a very precise view on the details of the electronic structure and increases the accuracy of model descriptions. We use the latter to calculate the Lindhard function and compare it with neutron scattering data. From the consistency of both, we conclude that the magnetic excitations and the AFQ propagation vector in CeB_6_ are dictated by the Fermi-surface geometry.

## Results

### Electronic structure from photoemission tomography

The method of choice to map the electronic structure in general is ARPES. But CeB_6_ holds several obstacles in store for this technique: it has a cubic crystal structure, that is, is fully 3D. This requires photon energy-dependent measurements to capture the *k*_*z*_ dispersion perpendicular to the surface. Moreover the material is hard and difficult to cleave, which is the usual procedure to measure single crystals. Third, hexaborides are subject to surface reconstructions and possess surface states, which might mask the bulk electronic structure^23^,^24^,^25^,^26^. Previous ARPES studies on CeB_6_ are therefore sparse^27^,^28^,^29^. The Fermi surface has also been studied by de-Haas–van Alphen measurements. The results are consistent with ellipsoids centred around the *X* points^30^.

**Figure 1:**
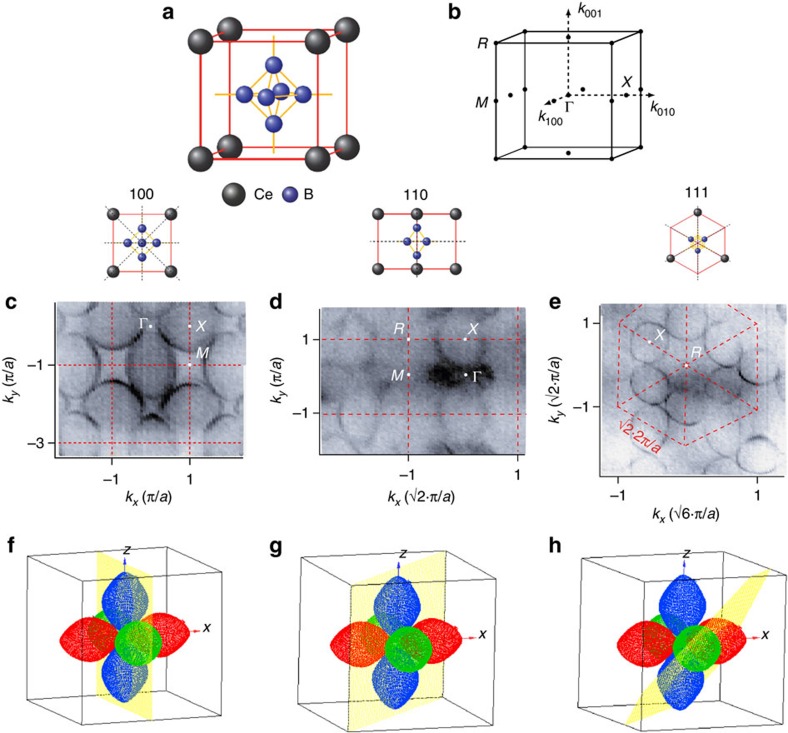
Fermi surface of CeB_6_ from different cleavage planes. (**a**) Crystal structure of CeB_6_. (**b**) Brillouin zone with high symmetry points. (**c**–**e**) Fermi surfaces and representations of the different cleavage planes. (**c**) (100), taken with a photon energy of *hv*=700 eV; (**d**) (110), *hv*=609 eV; (**e**) (111), *hv*=700 eV; measured at 12 K. (**f**–**h**) 3D representation of the measured Fermi surface and the measurement plane. The different colours of the ellipsoids are for clarity.

CeB_6_ crystallizes in a CsCl-type simple cubic crystal structure shown in Fig. 1a. The B_6_ octahedron is situated in a cubic environment of Ce atoms. In Fig. 1c–e, we present Fermi surface maps taken for different cleavage planes as indicated in the figure. The symmetry of the Fermi surface contours mirrors one of the cleavage plane. The (100) direction has a fourfold rotation axis, (110) only twofold and three- or sixfold for (111) depending on the used photon energy. The Fermi surface consists of ellipsoids centred at the *X* point. Figure 1f–h present 3D visualizations including the experimental cuts shown above. This Fermi surface agrees with previous measurements for CeB_6_ (refs 27, 28, 29, 30). The ellipsoids are typical for the hexaborides in general^28^,^29^. Their orbital character is composed of extended Ce 5*d* states with admixtures of localized Ce 4*f* near the Fermi energy (*E*_F_; ref. 28), similar to other 4*f* systems exhibiting a resonance mode^31^,^32^.

**Figure 2:**
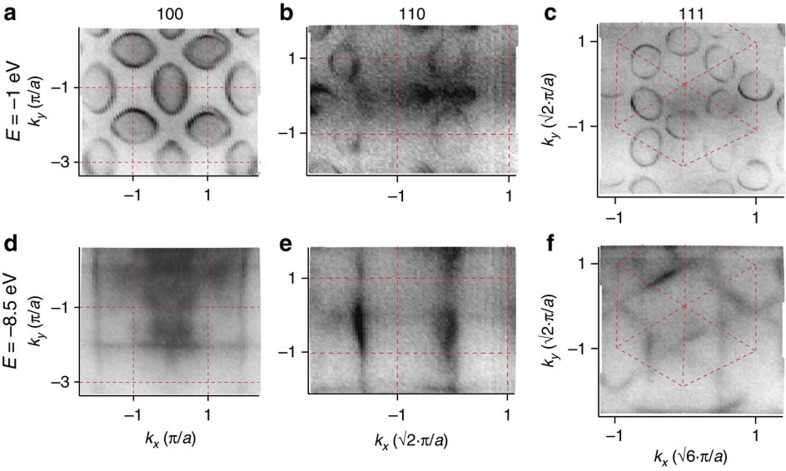
Constant energy cuts for different cleavage planes. (**a**–**c**) at *E*=−1 eV and (**d**–**f**) at *E*=−8.5 eV for (100) plane in (**a**,**d**), (110) in (**b**,**e**) and (111) in (**c**,**f**)

The ellipsoid bands are electron-like. Away from *E*_F_, their size shrinks (see Fig. 2). Far below the bottom of the ellipsoid band at *E*=−8.5 eV, the constant energy contour consists of straight sections reflecting the symmetry of the Brillouin zone (Fig. 2d–f).

**Figure 3:**
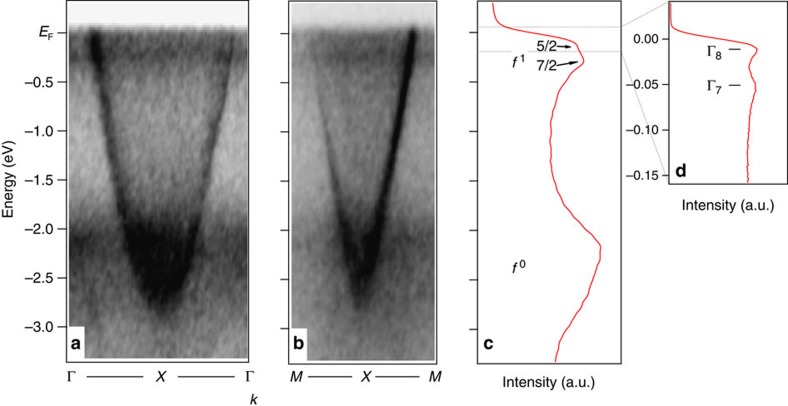
Band structure of CeB_6_. Energy distribution maps along (**a**) Γ*X* and (**b**) *MX* measured at 12 K. (**c**) *k*-integrated version of **a** with assignment of the *f*-levels. (**d**) The region near *E*_F_ measured separately with increased resolution at 1 K. Crystal field levels are denoted.

Figure 3a,b show the energy distribution maps along the two mirror axes of the ellipsoid in the (100) plane. Along the Γ*X* direction, the band has a parabola-like or U shape, whereas the bottom of the band appears more cusp-like or V shaped along *MX*. Figure 3c presents the **k**-integrated spectrum of panel 3a featuring the typical shape of Ce-based materials: Around *E*=−2.5 eV, the *f*^0^ ionization peak is situated, which overlaps with the bottom of the ellipsoidal band. Near *E*_F_, the screened *f*^1^ states are found, which split due to the spin–orbit coupling in a *J*=5/2 and 7/2 component. The 5/2 state at *E*_F_ is relevant here and splits further into crystal field levels, namely a Γ_7_ doublet and a Γ_8_ quartet. One of the Γ_8_ levels is occupied, whereas the Γ_7_ intensity seen in the spectrum is a satellite. The energy separation of the Γ_7_ and Γ_8_ levels (Δ*E*≈50 meV) is in agreement with previous reports^33^,^34^. Note that the large ground state degeneracy distinguishes CeB_6_ from many other Ce-based heavy fermion materials.

The electronic structure derived here by soft X-ray photoemission from various cleavage planes is consistent with previous low photon energy studies from the (100) plane^28^,^29^. Neupane *et al.* reported a strong **k**-dependent renormalization around the Γ point concluded from the deviation of the experimental data and the bandstructure calculation in this region and enhanced quasiparticle intensity. Our data also deviate from this calculation in a similar fashion but it is difficult to compare the intensity at the lowest energies due to the different integration windows imposed by the different energy resolution.

### Lindhard function and model description

**Figure 4:**
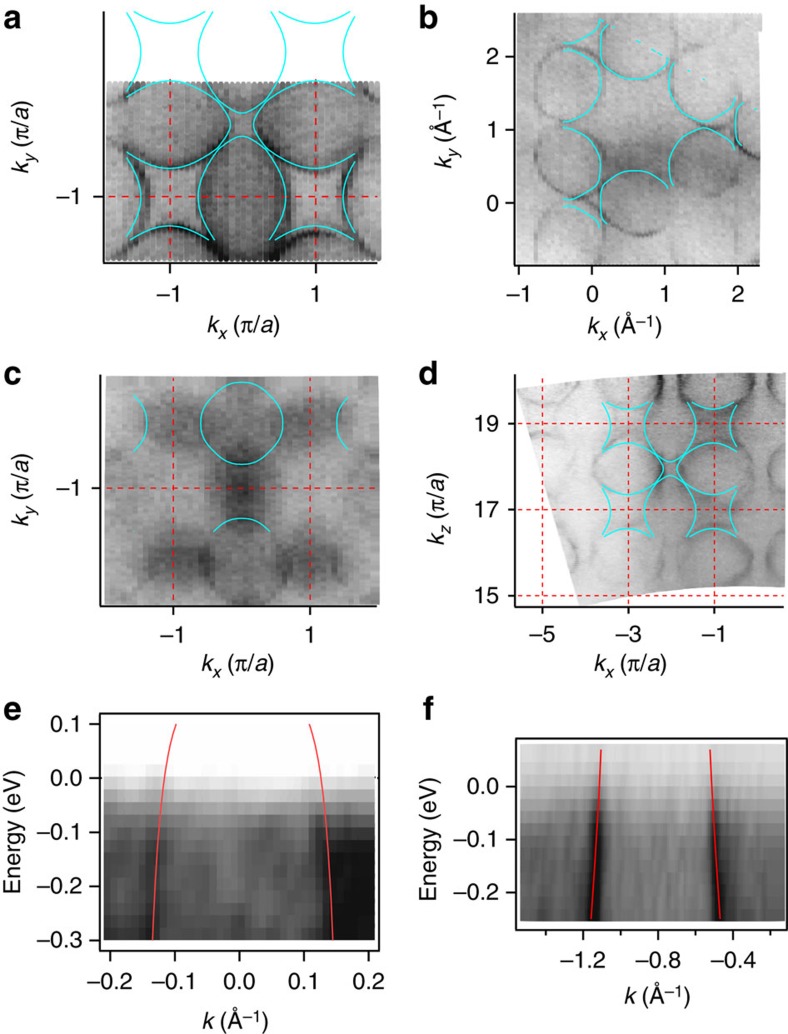
Parametrization of the low-energy electronic structure. Fermi surface for (**a**) (100), Γ plane; (**b**) (111), *R* plane; (**c**) (100), *R* plane; (**d**) (100), *k*_*z*_ scan; energy distribution maps for (**e**) (100), Γ*X* direction; (**f**) (100), *MX* direction.

To investigate the connection of the electronic structure with the magnetism and the AFQ phase, the experimental band structure has to be fitted to a suitable model which is then used to calculate the Lindhard function. In two-dimensional systems, nesting instabilities can be identified sometimes just by visual inspection of the Fermi surface. However, in three dimensions, this is not a viable procedure anymore and a rigorous treatment is required. For this purpose, we used a tight-binding-like model fitted to the experimental ARPES data. Figure 4a–f show the results of the fitting. Figure 4a,b compares the (100) and (111) Fermi surfaces to the model. Figure 4c presents the (100) Fermi surface at *k*_*z*_=*π*/*a*, where the ellipsoid is cut at 90° and gives almost a circular contour. The latter is superimposed by shadow-like structures arising from the nearby parts of the ellipsoids parallel to the cutting plane by remnant final state *k*_*z*_ broadening. Figure 4d shows a (100) *k*_*z*_ scan, that is, a photon energy-dependent measurement. In all cases, the experimental contours are well reproduced by the model. The same holds true for the comparison with the near *E*_F_ energy distribution maps in Fig. 4e,f. Note that the model electronic structure starts to deviate for *E*<−0.3 eV from the data. However, the Lindhard function falls off rapidly away from *E*_F_.

The condition for the formation of a spin-density wave (SDW) is approximately given by^16^:


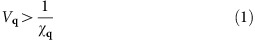


where *V*_**q**_ is the exchange interaction in the local approximation and *χ*_**q**_ is the real part of the Lindhard function in the static limit:


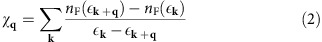


Here 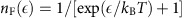 is the Fermi distribution. The system may become unstable against the formation of a SDW in the vicinity of maxima of *χ*_**q**_, that is, where the Fermi surface nesting is large.

### Inelastic neutron scattering

**Figure 5:**
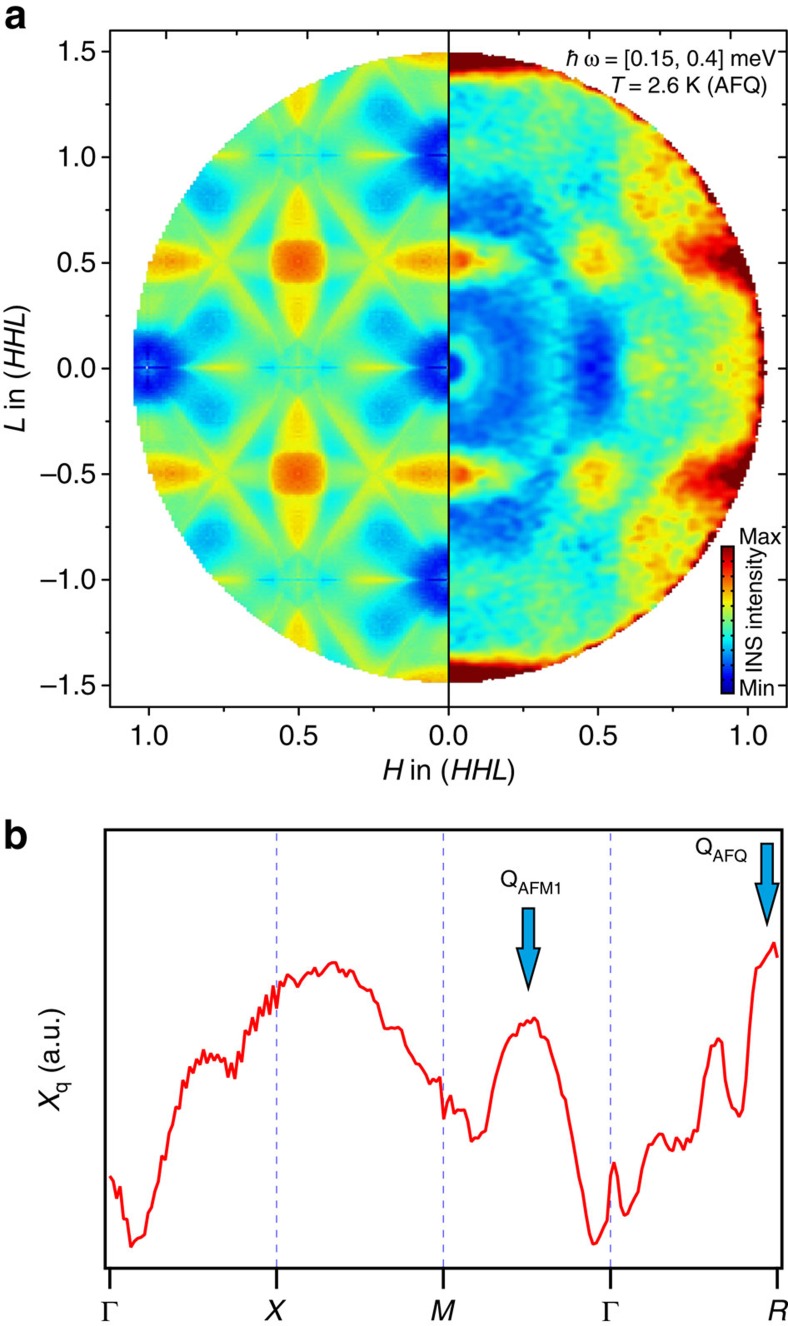
Comparison of Lindhard function and inelastic neutron scattering (INS) data. (**a**) Two-dimensional representation of the Lindhard function in the *(HHL)* plane compared with the distribution of magnetic quasielastic scattering intensity measured by INS at *T*=2.6 K>*T*_N_ in reciprocal lattice units. (**b**) Lindhard function extracted for certain high symmetry directions with the indication of peaks coinciding with the propagation vectors of low-temperature ordered phases.

The relevance of the calculated Lindhard function is confirmed by the experimental inelastic neutron scattering (INS) data as shown in Fig. 5a. The two pictures are remarkably similar. The calculation reproduces not only the maxima but also the qualitative shape of the magnetic diffuse scattering at *R* (1/2, 1/2, 1/2) [=(*π*, *π, π*)] and *X* (0, 0, 1/2) [=(0, 0, *π*)]. Moreover, line structures appear in the calculation (for example, from (1/2, 1/2, 0) to (1/4, 1/4, 1/2)), which resemble the oval-shaped streaks connecting the *R* points in the neutron data, resulting in a weak local maximum around the propagation vector of the AFM2 order, (1/4, 1/4, 1/2) [=(*π/*2, *π/*2, *π*)]. In Fig. 5b, *χ*_**q**_ is extracted along certain high symmetry directions. The maxima at **Q**_AFQ_ and **Q**_AFM1_ are clearly visible.

There is an additional broad maximum around *X* (0, 0, 1/2), which has a significant spectral weight but does not correspond to any static ordering^35^. This possibly indicates proximity to another AFM instability with a propagation vector (0, 0, 1/2), which loses the energy competition with multiple other competing order parameters (possibly due to its broad width in **Q**). In fact, this type of order is realized in Ce_1−*x*_ Nd_*x*_B_6_ for *x*>0.4 (ref. 36). But here also, both neutrons and ARPES are in good agreement.

## Discussion

**Figure 6:**
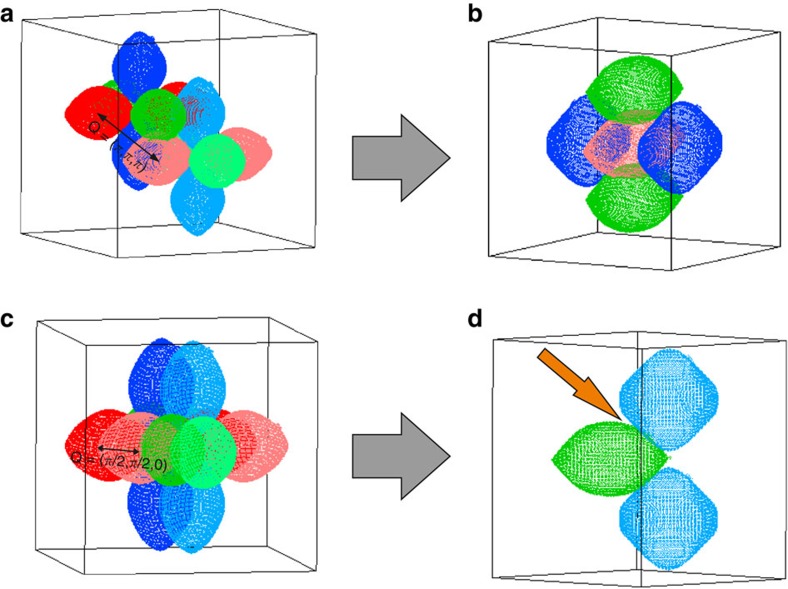
3D representations of the nesting conditions. (**a**,**c**) The original Fermi surface and their replicas by the respective nesting vector are shown. (**b**,**d**) Ellipsoids are skipped or added to emphasize the nesting condition. (*π*, *π, π*) in **a**,**b**; (*π/*2, *π/*2, 0) in **c**,**d**. The arrow highlights the place of increased nesting.

The agreement between the Lindhard susceptibility derived from the measured electronic structure and the INS data proves the itinerant character of the magnetic excitations in CeB_6_ and suggests that the propagation vector of the AFQ order is dictated by the Fermi-surface geometry. It is important to emphasize that this result does not contradict the AFQ nature of the hidden-order phase, as the itinerant electrons determine the RKKY interactions between the Ce 4*f* multipolar moments that can be still considered as local. To visualize the nesting condition, we show in Fig. 6 the 3D Fermi surface together with shifted replicas. Figure 6a presents the nesting by (*π*, *π, π*). This vector effectively shifts a given ellipsoid into the void formed in between the other four. This maximizes the overlap among the ellipsoids although nesting in the strict sense is not apparent. Figure 6b shows the (*π/*2, *π/*2, 0) vector. Here, clear nesting between parallel segments of the Fermi surface is revealed.

Primordial signatures of the complex magnon spectrum of CeB_6_ are already observed above *T*_N_ in the quasielastic neutron scattering response^35^. We show here that these signatures have their natural explanation in the low-energy electronic structure, establishing the importance of itinerant electrons for the spin dynamics. For CeB_6_, a picture emerges where the propensity towards specific magnetic order roots in favourable nesting conditions of the Fermi surface. The exact way by which this is achieved could be quite complex. For example, a weak magnetic Bragg peak has been observed at **Q**_AFQ_ above *T*_N_ (refs 15, 37). It has been ascribed to a SDW-type order associated with the conduction electrons primarily of Ce 5*d* character^37^. (Note that the AFQ order itself is hidden to neutron diffraction^10^.) Although orders of magnitude weaker than the antiferromagnetic order observed below *T*_N_, this itinerant SDW could break the degeneracy of the Γ_8_ crystal field ground state and promote the multipolar moments to order in the AFQ phase with the same propagation vector. The value of *T*_Q_ should then be associated with the low temperature evolution of the electronic structure, for example, the mass enhancement due to the formation of heavy quasiparticles. The latter could lead to an enhancement of the nesting-related energy gain. Intriguingly, this is indeed observed, as *T*_Q_ coincides in zero field with the formation of the heavy fermion liquid indicated by the drop in resistivity^38^. Another plausible scenario would involve an enhancement of the RKKY interaction between the multipolar moments^18^ at the *R* point due to the Fermi-surface nesting, analogous to that seen previously in rare-earth silicides^17^.

The ellipsoidal Fermi surface observed here for CeB_6_ is also typical for other rare earth hexaborides, for example, the presumed topological Kondo insulator SmB_6_ (refs 39, 40, 41, 42). Interestingly, recent INS data of SmB_6_ bear out similarities to CeB_6_ as well, namely intensity maxima at the *X* and *R* points, for which Fermi surface nesting was discussed^43^. Our explicit observation of the relevance of nesting in CeB_6_ strongly indicates that the same mechanism is operative in SmB_6_ too.

Multipolar ordering phenomena have been suggested for other hidden order compounds, most prominently URu_2_Si_2_, which has so far even resisted proper characterization by conventional solid state probes^1^. CeB_6_, on the other hand, is well characterized with regard to its low-temperature ordered phases and by now also to its electronic structure and the connection between both. Therefore, it may serve as a model compound for many heavy-fermion metals and thus help to elucidate the interplay between the electronic structure, itinerant magnetism and complex order in correlated electron systems in general.

Moreover our methodology, namely the precise modelling of the 3D electronic dispersion based on experimental ARPES data and subsequent usage of this model to calculate complex response functions, is applicable to a wide class of 3D materials, not restricted to *f*-systems.

## Methods

### Sample preparation

Single crystal samples of CeB_6_ were prepared by floating zone method as described in ref. 15. Before the measurement, the crystals were oriented by Laue diffraction, and on the outside of the sample small notches were cut to create a predetermined cleavage plane along the desired direction. After that, the samples were cooled in ultrahigh vacuum with a base pressure of 1 × 10^−10^ mbar by a He flow cryostat to 12 K and cleaved for the measurement.

### ARPES measurements

ARPES measurements have been done at the SX-ARPES end station of the Swiss Light Source^44^, ADRESS beamline^45^ in the photon energy range of 500–900 eV. The energy resolution was set to Δ*E*=100 meV. Angle resolution was better than 0.07°. The high resolution measurement of the crystal field splitting in Fig. 3d has been carried out at the 1^3^ beamline at BESSY with Δ*E*=10 meV at a temperature of *T*=1 K with a photon energy of *hv*=90 eV.

### INS measurements

INS data were collected using the cold-neutron time-of-flight spectrometer IN5 at the high-flux research reactor of the Institute Laue-Langevin^35^,^46^. A large single crystal specially synthesized from isotope-enriched ^11^B to minimize neutron absorption was mounted in a standard cryostat with its (110) and (001) directions in the horizontal plane. The incident neutron wavelength was fixed at 5 Å (3.27 meV), yielding an energy resolution (full width at half maximum) of 0.08 meV at zero energy transfer. The measurements were taken while rotating the sample about the vertical axis and then combined and transformed into energy-momentum space using HORACE analysis software. The quasielastic intensity distribution shown in Fig. 5a was obtained by integrating the four-dimensional data set in a narrow slab parallel to the horizontal plane along the vertical direction of the momentum and in a broad energy window between 0.15 meV (immediately above the elastic line) and 0.4 meV, thus providing an estimate of the integral quasielastic spectral weight within the *(HHL)* plane.

### Model description

The low energy electronic structure has been parametrized by the following tight-binding-like model:


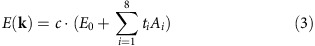


Where *t*_*i*_ are hopping parameters, *A*_*i*_ tight-binding expansions of the *i-*th order and *c* is a correction term with


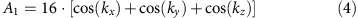



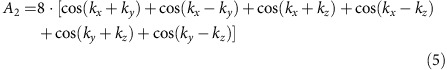



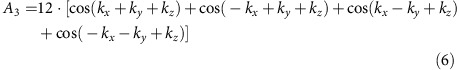



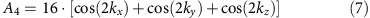



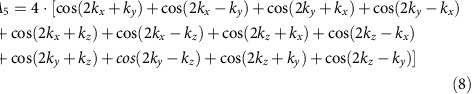



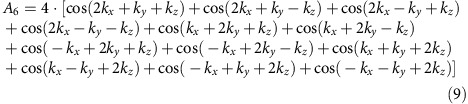



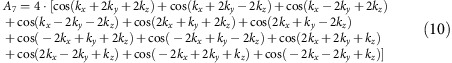



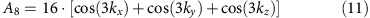


with 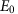
=0.215 eV, 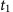
=−0.091 eV, 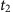
=0.118 eV, 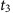
=0.0085, eV, 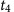
=−0.011 eV, 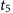
=−0.089 eV, 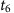
=0.077 eV, 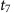
=−0.0039, eV, 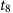
=−0.011 eV; and


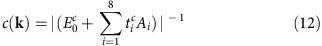


with 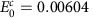, 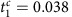, 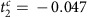, 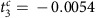, 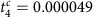, 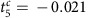, 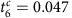, 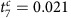, 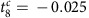. The model is only valid for *E*≥−0.3 eV.

### Software availability

The open-source MATLAB-based HORACE software package is available from http://horace.isis.rl.ac.uk.

## Additional information

**How to cite this article:** Koitzsch, A. *et al.* Nesting driven multipolar order in CeB_6_ from photoemission tomography. *Nat. Commun.* 7:10876 doi: 10.1038/ncomms10876 (2016).
